# Evaluation of common variants in *MG53* and the risk of type 2 diabetes and insulin resistance in Han Chinese

**DOI:** 10.1186/s40064-016-2218-1

**Published:** 2016-05-12

**Authors:** Song Yang, Hailong Zhao, Kuangfeng Xu, Yun Qian, Ming Wu, Tao Yang, Yanchun Chen, Xianghai Zhao, Jinfeng Chen, Jinbo Wen, Zhibing Hu, Harvest F. Gu, Hongbing Shen, Chong Shen

**Affiliations:** 1 Department of Cardiology, Affiliated Yixing People’s Hospital of Jiangsu University, People’s Hospital of Yixing City, Yixing, 214200 China; 2 Department of Epidemiology, School of Public Health, Nanjing Medical University, 101 Longmian Avenue, Jiangning, Nanjing, 211166 China; 3 Department of Endocrinology, The First Affiliated Hospital with Nanjing Medical University, Nanjing, 210029 China; 4 Department of Chronic Non-communicable Disease Control, Wuxi Center for Disease Control and Prevention, Wuxi, 214023 China; 5 Institute of Chronic Disease Control, Center for Disease Control and Prevention of Jiangsu Province, Nanjing, 210009 China; 6 Department of Clinical Epidemiology, Jiangsu Province Geriatrics Institute, Nanjing, 210024 China; 7 Department of Molecular Medicine and Surgery, Rolf Luft Research Center for Diabetes and Endocrinology, Karolinska University Hospital, Karolinska Institutet, 17176 Stockholm, Sweden

**Keywords:** MG53, Type 2 diabetes, Insulin resistance, Insulin sensitivity, Genetic association

## Abstract

Abnormally increased skeletal-muscle-specific E3 ubiquitin ligase (MG53) is associated with the inhibition of insulin signalling and insulin resistance (IR) in animal models. Four community-based studies of Han Chinese populations were included in this study to test the association of variants of *MG53* and type 2 diabetes (T2D). The results showed that rs7186832 and rs12929077 in MG53 were significantly associated with T2D and impaired fasting glucose (IFG) of females in the discovery-stage case–control study and cohort study respectively of rural population but not in the replication sample of urban population. In rural population, the fasting insulin (mU/L) of the subjects with AA, AG and GG genotypes in rs12929077 were 8.70 ± 8.05, 10.71 ± 11.16 and 13.41 ± 14.26, respectively, and increased linearly in T2D cases without medication treatment (P = 0.04). This variant was significantly associated with HOMA-IR (P = 0.020) and HOMA-IS (P = 0.023). In individuals with IFG, the insulin and HOMA-IR of AG carriers were significantly higher than those of AA carriers. In urban population, after glucose loading, there were significant differences in the 30-min glucose, the area under the curve (AUC) of 30-min glucose and the AUC of 120-min glucose according to the genotypes of rs7186832 and rs12929077 in males but not females. Our findings suggest that MG53 variants might confer risk susceptibility to the development of T2D of females and IR particularly in rural population.

**Electronic supplementary material:**

The online version of this article (doi:10.1186/s40064-016-2218-1) contains supplementary material, which is available to authorized users.

## Background

Diabetes affects approximately 10 % of the world’s adult population and is one of the leading risk factors for cardiovascular disease, renal failure and visual impairment (Shaw et al. 2010; van Dieren et al. 2010). Of all of the categories of diabetes, type 2 diabetes mellitus (T2D) accounts for approximately 90 %. In recent decades, the prevalence of T2D has increased rapidly due to ageing of the population, urbanization and lifestyle changes, making it one of the most important public health challenges in China (Xu et al. 2008; Wong and Wang 2006). Although the increase in T2D prevalence is caused by environmental factors, there is considerable evidence that T2D is highly heritable (Pyke 1979; Sandler 1984; Jirkovska 1989). Genome-wide association studies have identified a number of susceptibility loci associated with T2D (Tsai et al. 2010; Sladek et al. 2007; Scott et al. 2007). However, these loci account for only some of the genetic variants in T2D, suggesting that much remains to be discovered. Further research should reveal additional genetic factors based on the understanding of the mechanisms that are involved in the development of T2D.

Insulin resistance (IR), defined as decreased glucose uptake and disposal ability, along with defects in insulin secretion, are fundamental elements in the aetiology of T2D. In the early stage of the disease, IR is highest in skeletal muscle (Kahn 1994), which accounts for approximately two-thirds of glucose utilization after meals. Several studies have demonstrated that insulin receptor, insulin receptor substrate 1 (IRS1) and kinase activities are decrease in the muscles of early-T2D patients (Goodyear et al. 1995; Caro et al. 1987), suggesting that insulin receptor and IRS1 play important roles in the insulin signalling pathway (Hepp 1980). Further studies have shown that insulin binds insulin receptor and then activates IRS protein tyrosine phosphorylation immediately after initiating insulin’s downstream effects, including the activation of phosphatidylinositol 3-kinase (PI3K) and the translocation of glucose transporter 4 (Frattali et al. 1991; Murakami and Rosen 1991; Ma et al. 2013). In contrast to the glucose tolerance change observed in IRS1 tissue-specific knockout mice (Bruning et al. 1998), animals with muscle-specific insulin receptor knockout exhibited features of T2D without a change in glucose tolerance. Therefore, IR in skeletal muscle may be a key player in the development of T2D, but the mechanisms that are involved in skeletal muscle IR remain uncertain.

Recently, a report published in Nature has proposed a new mechanism underlying IR in skeletal muscle and metabolic syndrome, identifying a novel role of Mitsugumin 53 (MG53) as a muscle-specific E3 ubiquitin ligase targeting insulin receptor and IRS1 (Song et al. 2013). The specific inhibition of the E3 ubiquitin ligase of MG53 prevents the degradation of insulin receptor and IRS1 and indicate that MG53, acting as an E3 ubiquitin ligase, is a key negative regulator of insulin signal in skeletal muscle and that the overexpression of MG53 confers a risk of metabolic disorders (Song et al. 2013). This evidence provides important new insight into the pathophysiology of T2D, but there is no report to date of evidence for this association in a human population. Herein, we investigated the association between *MG53* polymorphisms and T2D and IR in four community-based studies in Han Chinese populations.

## Methods

### Subjects

In the primary stage (discovery phase), our case–control study (Nantong population) consisted of 776 T2D patients (249 men/527 women), 522 IFG subjects (180 men/342 women) and 957 NGT control subjects (331 men/626 women) who were recruited to find positive genetic variants of MG53 for T2D. The participants were selected from a rural population of 14,469 subjects in two towns east of the Nantong City, Jiangsu province in 2008. According to the diabetes diagnosis criteria of the America Diabetes Association (ADA), IFG and T2D are defined by fasting plasma glucose (FPG) levels 5.6–7.0 and ≥7.0 mmol/L, respectively, or having a self-reported T2D history, and NGT is defined by a normal FPG of ≤5.6 mmol/L. A questionnaire including age, gender, nationality, education level, physical activity and household income was given to participants to gather demographic characteristics. The interview also included questions associated with the diagnosis and treatment of diabetes, hypertension, dyslipidemia and cardiovascular events. Weight, height, blood pressure and waist circumference were measured twice by different trained staff members. BMI was calculated as the weight (in kilograms) divided by the square of the height (in metres). A simplified version of the international physical activity questionnaire (IPAQ) was applied to evaluate PAI by self-reporting of 24-h physical activity. The PAI was calculated based on the hours and metabolic energy estimate (MET) of physical activities, including sleeping (1 MET), watching TV or sitting (1.1 MET), light activity (1.5 MET), moderate activity (4 MET), and vigorous activity (8 MET).

The further replication stage consisted of three study populations, also from Jiangsu province including a case–control study of T2D in an Wuxi City urban population, a baseline survey for cardiovascular disease cohort study of a general rural population in Yixing City and further average 5.18 years follow-up (from May, 2014 to October, 2015) was carried out. A cross-sectional study of an IFG and impaired glucose tolerance (IGT) population subjected to an oral glucose tolerance test (OGTT) from the program of metabolic disease surveys in an urban population of Gulou District, Nanjing City. Similarly to the data acquired from the Nantong population, demographic information, disease history, family history of diabetes, behaviour profiling and anthropometric variables, except physical activity, were acquired.

The replication case–control study of the Wuxi population was based on a community epidemiological survey, and unrelated ethnic Han Chinese individuals aged more than 30 years were enrolled. A total of 1200 T2D cases defined as above and 1200 age- and gender-matched healthy controls were included.

HOMA indices, including HOMA-IR, HOMA-IS and HOMA-β, which were used to assess IR, insulin sensitivity and the function of islet β cells, respectively, were estimated from serum insulin and fasting glucose in 4222 adults aged 30 years or more in the Yixing cohort study. This population included 497 T2D cases, 875 subjects of IFG and 2850 subjects of NGT defined as above.

During the community chronic disease survey in Gulou District, Nanjing City, all of the subjects aged more than 40 years who were free of diabetes after fasting glucose and 2-h postprandial glucose detection accepted an OGTT (75 g of glucose) and venous blood samples drew at 0, 30 and 120 min were used to access IR and pancreatic islet β cell function by calculating the AUCs of fasting glucose and insulin. Finally, 1932 individuals were diagnosed with prediabetes, and the numbers of IFG, IGT (2-h blood glucose was 7.8–11.1 mmol/L after 75 g of glucose OGTT) and both IFG and IGT were 329, 1322 and 281, respectively.

The methods were approved by the Nanjing Medical University (Nanjing) and were carried out in accordance with the approved relevant guidelines. All of the individuals provided written informed consent before participation in the study.

### Sequencing analysis of *MG53*

*MG53* (gene ID: 493829), located on human chromosome 16p11.2 and spanning 14,519 bp, is conserved in animals. To discover single nucleotide polymorphisms (SNPs) of MG53, 20 Chinese healthy control subjects and 30 extreme phenotype patients of BMI > 30 and fasting blood glucose >10 mmol/L were selected for *MG53* sequencing analysis by the Sanger sequencing method. Twelve pairs of primers covering the entire region of *MG53,* as well as regions 2 k upstream and 1 k downstream, were designed for amplification and sequencing analysis on the basis of GenBank sequences (Ref. Seq. of *MG53* NC_000016). No other variant was identified different from the International HapMap Project (HapMap Data Rel 24/phase II Nov08, on NCBI B36 assembly, dbSNP b126). Thus, two tagSNPs rs7186832 and rs12929077 were selected and further genotyped in this study (Additional file 1: Figure S1).

### DNA isolation and genotyping

Blood samples were collected in K_3_-EDTA tubes. Proteinase K digestion and phenol–chloroform extraction were used to isolate genomic DNA from whole blood. Finally, DNA was purified and diluted to 10 ng/μL. Genotyping was performed using TaqMan technology and a 7900HT Fast Real-Time PCR System (Applied BioSystems, Foster City, CA, USA). A 5-μl reaction mixture consisting of 10 ng of DNA, 2.4 μL of TaqMan universal PCR master mix, 0.1 μL of forward and reverse primers and FAM and VIC probes, and 1.2 μl of H_2_O were prepared for PCR reaction. The TaqMan-MGB probes and primers were all ordered from Applied BioSystems. The genotype was determined automatically with the Sequence Detection System 2.1 software (95 % autocaller confidence level). Then, 5 % of the samples were repeated to determine the consistency, which was greater than 99.5 %. The genotype-calling success rates were greater than 99.9 %.

### Calculations

Homeostasis model assessment (HOMA-IR, HOMA-β and HOMA-IS) was used to evaluate IR, insulin secretion and insulin sensitivity. These values were calculated using the following equations: HOMA-IR = fasting plasma glucose × fasting plasma insulin/22.5, HOMA-β=20 × fasting plasma insulin/ (fasting plasma glucose-3.5), and HOMA-IS = 1/(fasting plasma insulin × fasting plasma glucose). The unit of fasting plasma glucose was mmol/L, and the unit of fasting plasma insulin was mU/mL. The ratio of insulin increment to FPG increment 30 min after glucose loading (ΔI30/ΔG30, insulin:glucose ratio, IGR) (Seltzer et al. 1967), area under the curves (AUCs) for glucose and insulin 30 and 120 min after glucose loading, and the ratio of AUC of insulin and the AUC of glucose (insulin release index, IRI) (Stumvoll et al. 2000) were calculated to further evaluate the first-stage islet secretory responses to glycaemic stimulus.

### Statistical analysis

A Kolmogorov–Smirnov test was used to determine the normality of distribution of quantitative variables. The quantitative variables of non-normal distributions were natural logarithm transformed to obtain normal distributions for statistical analysis. Levene’s test was performed for homogeneity testing. Fisher’s exact test was used to test for HWE in the NGT control group. The genotype and allele distribution between cases (T2D and/or IGT subjects) and NGT control individuals were compared using Chi squared (χ^2^) tests. A comparison of quantitative variables between different genotypes was performed with ANOVA, and a general linear regression was used to adjust for covariates. An ordinal multinomial logistic regression model was performed to estimate the risk of T2D with or without adjustment for age, gender, BMI, or PAI. Bonferroni correction was used for multiple comparisons. The odds ratio (OR) and 95 % confidence intervals (CI) were used to test for association in case–control study and the hazard ratio (HR) and 95 % CI by Cox regression were used to estimate the risk of IFG and T2D in cohort study. All of the statistical analyses were performed with SPSS version 15.0 (SPSS, Inc., Chicago, USA). A two-tailed P < 0.05 was considered statistically significant.

## Results

### Clinical characteristics

The demographic and clinical characteristics of the four studied populations are summarized in Table 1. Age (5 years) and gender matching were performed in the Nantong, Wuxi and Yixing populations. The indices of body mass index (BMI), systolic blood pressure (SBP), diastolic blood pressure (DBP), waist circumference, fasting plasma glucose (FPG), fasting plasma insulin (FINS), homeostasis model assessment (HOMA)-IR and HOMA-IS in the normal glucose tolerance (NGT), impaired fasting glucose (IFG) and T2D groups increased linearly, and the physical activity index (PAI) and HOMA-β decreased linearly (P_trend_ < 0.05).

**Table 1:** Clinical characteristics of NGT, IFG, and T2D subjects in Nantong, Wuxi, Yixing and Gulou populations

Characteristics	Nantong population			Wuxi population		Yixing population			Gulou
	NGT (n = 957)	IFG (n = 522)	T2D (n = 776)	NGT (n = 1200)	T2D (n = 1200)	NGT (n = 2850)	IFG (n = 875)	T2D (n = 497)	Prediabetes (n = 1932)
Female (n %)	626 (65.41 %)	342 (65.52 %)	527 (67.91 %)	722 (60.17)	722 (60.17 %)	1685 (59.12 %)	535 (61.14 %)	287 (57.74 %)	1244 (64.39 %)
Age (years)	58.54 ± 9.47	58.05 ± 9.95	58.17 ± 8.56	56.43 ± 8.02	57.43 ± 9.77	60.33 ± 10.71	60.86 ± 10.88	61.75 ± 10.30^*^	58.64 ± 9.26
BMI (kg/m^2^)	21.31 ± 1.50	25.75 ± 3.85	25.54 ± 3.55^**^	22.64 ± 2.86	24.92 ± 3.41**	23.83 ± 3.31	24.82 ± 3.44	25.08 ± 3.47^**^	25.07 ± 3.32
Waist circumference (cm)	75.49 ± 6.33	88.22 ± 10.43	88.96 ± 10.35^**^	81.42 ± 9.48	85.41 ± 9.23	83.51 ± 9.09	86.37 ± 9.34	86.83 ± 9.36^**^	
Systolic BP (mmHg)	113.79 ± 11.45	130.43 ± 19.14	132.72 ± 19.52^**^	118.17 ± 14.88	137.47 ± 21.20	132.02 ± 15.65	135.43 ± 15.60	135.39 ± 16.90^**^	132.61 ± 16.77
Diastolic BP (mmHg)	68.28 ± 7.90	77.46 ± 11.10	78.11 ± 10.86^**^	75.83 ± 8.64	80.12 ± 10.25	82.70 ± 8060	84.13 ± 8.53	83.64 ± 8.97^**^	78.94 ± 10.47
PAI	63.46 ± 19.99	60.43 ± 20.68	53.81 ± 20.91^**^	–	–	–	–	–	–
FPG (mmol/L)	4.18 ± 0.4618	6.08 ± 0.37	8.44 ± 3.31^**^	4.51 ± 0.46	8.97 ± 3.52	4.89 ± 0.59	6.04 ± 0.36	9.52 ± 3.24^**^	5.80 ± 0.51
FINS (mU/L)	–	–	–	–	–	5.74 ± 4.39	7.36 ± 6.08	9.65 ± 5.74^**^	12.51 ± 7.76
HOMA-β	–	–	–	–	–	81.70 ± 260.99	58.47 ± 46.90	41.91 ± 54.71^**^	113.36 ± 75.85
HOMA-IR	–	–	–	–	–	1.26 ± 0.99	1.98 ± 1.68	4.16 ± 4.49^**^	3.24 ± 2.07
HOMA-IS	–	–	–	–	–	0.07 ± 0.10	0.04 ± 0.04	0.02 ± 0.03^**^	0.02 ± 0.016

*n* number of subjects, *BMI* body mass index, *PAI* physical activity index, *FPG* fasting plasma glucose, *FINS* fasting insulin, *HOMA* homeostasis model assessment, *IR* Insulin resistance, *NGT* normal glucose tolerance, *IFG* impaired fasting glucose, *P* probability, *T2D* type 2 diabetes

* Significant differences in characteristics between NGT, IGT and T2D group (* P < 0.05, ** P < 0.0001) were determined by two-tailed Student’s *t* test for quantitative data (mean ± standard deviation) and two-sided Chi squared test for categorical data (%). HOMA-β and HOMA-IR are log-transformed to follow normal distribution for comparison

### Genetic association analyses of case–control study

In Nantong population, both rs7186832 and rs12929077 were confirmed to be in Hardy–Weinberg equilibrium (HWE) in the control group (P > 0.05). As shown in Table 2, the additive (TT vs TC vs CC) and dominant (TT vs TC + CC) models of rs7186832 were significantly associated with T2D in the Nantong population after adjusting for BMI, age, gender and PAI. The ORs (95 % CIs) were 1.31 (1.10–1.57) and 1.21 (1.03–1.40), and the P values were 0.014 and 0.003, respectively. After Bonferroni correction, the dominant model of rs7186832 remained statistically significant (P = 0.003 × 6). The AG and GG genotypes (dominant) of rs12929077 carrier showed significant risk of T2D than AA genotype carrier and the OR (95 % CI) was 1.18 (1.02–1.39), P = 0.049, after adjustment for age, gender, BMI and PAI.

**Table 2:** Associations of *MG53* rs7186832 and rs12929077 with T2D in Nantong population

SNP	Group	WT/Ht/MT	Additive model (WT vs Ht vs MT)			Dominant model (WT vs Ht + MT)		
			OR (95 % CI)^a^	OR (95 % CI)^b^	OR (95 % CI)^c^	OR (95 % CI)^a^	OR (95 % CI)^b^	OR (95 % CI)^c^
rs7186832		TT/TC/CC						
	NGT	637/282/38	1.03 (0.89–1.18)	1.03 (0.89–1.18)	1.21 (1.03–1.40)	1.06 (0.91–1.24)	1.07 (0.91–1.26)	1.31 (1.1–1.57)
	IFG	360/142/20	P = 0.717	P = 0.680	P = 0.014	P = 0.489	P = 0.439	P = 0.003
	T2D	502/247/26						
rs12929077		AA/AG/GG						
	NGT	570/343/41	0.99 (0.86–1.13)	0.99 (0.87–1.14)	1.13 (0.98–1.32)	1.01 (0.86–1.17)	1.02 (0.87–1.19)	1.18 (1.02–1.39)
	IFG	320/182/16	P = 0.850	P = 0.070	P = 0.089	P = 0.968	P = 0.825	P = 0.049
	T2D	459/283/29						

*SNP* single nuclear polymorphisms, *WT* wild type, *Ht* heterozygote, *MT* mutant type, *OR* odds ratio, *CI* confidence interval, *NGT* normal glucose tolerance, *IFG* impaired fasting glucose, *P* probability, *T2D* type 2 diabetes

^a^P value of χ^2^ test for comparison of genotype between case and control groups

^b^Ordinal multinomial logistic regression analysis adjusted for age, gender and PAI

^c^Ordinal multinomial logistic regression analysis adjusted for age, gender, BMI and PAI

Further stratification analysis by gender showed that the dominant models of rs7186832 and rs12929077 were significantly associated with T2D in the female population, and the ORs (95 % CIs) were 1.58 (1.27–1.96) and 1.34 (1.08–1.65) with P values of 3.9 × 10^−5^ and 0.006, respectively (Table 3). The association strength of the two SNPs with T2D in females was even higher than that in the whole population, whereas no association was found in the males.

**Table 3:** Stratification analysis of *MG53* rs7186832 and rs12929077 with T2D in Nantong population

SNP	Gender	Group	WT/Ht/MT	Additive model (WT vs Ht vs MT)			Dominant model (WT vs Ht + MT)		
				OR (95 % CI)^a^	OR (95 % CI)^b^	OR (95 % CI)^c^	OR (95 % CI)^a^	OR (95 % CI)^b^	OR (95 % CI)^c^
rs7186832			TT/TC/CC						
	Male	NGT	204/115/12	0.83 (0.65–1.05)	0.83 (0.65–1.06)	0.96 (0.74–1.24)	0.75 (0.57–0.99)	0.77 (0.58–1.02)	0.91 (0.67–1.23)
		IGT	125/47/8	P = 0.121	P = 0.136	P = 0.743	P = 0.049	P = 0.071	P = 0.538
		T2D	171/67/10						
	Female	NGT	433/167/26	1.15 (0.97–1.36)	1.14 (0.97–1.36)	1.36 (1.12–1.63)	1.25 (1.03–1.53)	1.25 (1.03–1.54)	1.58 (1.27–1.96)
		IGT	235/95/12	P = 0.114	P = 0.177	P = 0.001	P = 0.024	P = 0.026	P = 3.9 × 10^−5^
		T2D	331/180/16						
rs12929077			AA/AG/GG						
	Male	NGT	184/133/14	0.84 (0.66–1.06)	0.83 (0.65–1.05)	0.94 (0.73–1.22)	0.80 (0.61–1.04)	0.80 (0.61–1.05)	0.82 (0.67–1.24)
		IGT	111/63/5	P = 0.138	P = 0.124	P = 0.641	P = 0.107	P = 0.112	P = 0.580
		T2D	154/85/10						
	Female	NGT	386/210/27	1.07 (0.91–1.27)	1.09 (0.92–1.28)	1.24 (1.03–1.47)	1.12 (0.93–1.39)	1.14 (0.94–1.39)	1.34 (1.08–1.65)
		IGT	209/119/11	P = 0.407	P = 0.329	P = 0.019	P = 0.225	P = 0.172	P = 0.006
		T2D	305/198/19						

*SNP* single nuclear polymorphisms, *WT* wild type, *Ht* heterozygote, *MT* mutant type, *OR* odds ratio, *CI* confidence interval, *NGT* normal glucose tolerance, *IFG* impaired fasting glucose, *P* probability, *T2D* type 2 diabetes

^a^P value of χ^2^ test for comparison of genotype between case and control groups

^b^Ordinal multinomial logistic regression analysis adjusted for age, gender and PAI

^c^Ordinal multinomial logistic regression analysis adjusted for age, gender, BMI and PAI

A further assessment of the association of rs7186832 or rs12929077 and T2D was performed in the Wuxi population, in which the association was not significant in this population and stratification analysis by gender (P > 0.05, Table 4).

**Table 4:** Associations analysis of MG53 rs7186832 and rs12929077 with T2D in Wuxi population

SNP	Gender	Group	WT/Ht/MT	Additive model (WT vs Ht vs MT)		Dominant model (WT vs Ht + MT)	
				OR (95 % CI)	P value	OR (95 % CI)	P value
rs7186832	Whole population	Control	756/392/50				
		T2D	738/391/62	1.03 (0.89–1.20)	0.61	1.02 (0.85–1.21)	0.81
	Male	Control	289/169/19				
		T2D	286/159/30	1.03 (0.82–1.30)	0.75	0.97 (0.73–1.28)	0.84
	Female	Control	467/223/31				
		T2D	452/232/32	1.04 (0.86–1.26)	0.63	1.06 (0.84–1.33)	0.59
rs12929077	Whole population	Control	726/409/56				
		T2D	716/415/69	1.04 (0.90–1.21)	0.51	1.04 (0.87–1.24)	0.65
	Male	Control	278/175/21				
		T2D	277/167/34	1.05 (0.84–1.32)	0.64	0.99 (0.75–1.31)	0.96
	Female	Control	448/234/35				
		T2D	439/248/35	1.05 (0.87–1.26)	0.60	1.08 (0.863–1.35)	0.49

P value of Logistic regression for comparison of genotype between case and control groups adjusted for BMI, age and gender

*SNP* single nuclear polymorphisms, *WT* wild type, *Ht* heterozygote, *MT* mutant type, *OR* odds ratio, *CI* confidence interval, *P* probability, *T2D* type 2 diabetes

### Genetic association analyses of cohort study

In Yixing population, 3490 subjects (84.5 %) were followed, 364 (12.7 %) subjects with NGT were developed to IFG and 53 (1.9 %) were developed to T2D. 110 (12.1 %) subjects with IFG were developed to T2D. Cox regression analyses showed that there was no significant association detected between rs7186832 or rs12929077 and the risk of T2D developing from NGT or IFG (Additional file 1: Table S1). The variation of TC + CC (vs. TT) of rs7186832 and AG/GG (vs. AA) were significantly associated with the risk of IFG developing from NGT in females but not in males after adjustment for age and BMI, and the HRs (95 % CIs) were 1.556 (1.184–2.045) and 1.481 (1.127–1.948) with P values of 0.002 and 0.005, respectively (Table 5).

**Table 5:** Association of MG53 genotypes with the risk of IFG from NGT in Yixing cohort population

SNPs	Gender	Additive model		Dominant model	
		HR (95 % CI)^a^	P^a^	HR (95 % CI)^a^	P^a^
rs7186832	Whole population	1.077 (0.899–1.291)^a^	0.421^a^	1.192 (0.967–1.47)^a^	0.1^a^
	Male	0.821 (0.606–1.112)^b^	0.202^b^	0.819 (0.584–1.148)^b^	0.247^b^
	Female	1.283 (1.022–1.611)^b^	0.032^b^	1.556 (1.184–2.045)^b^	0.002^b^
rs12929077	Whole population	1.035 (0.869–1.234)^a^	0.698^a^	1.116 (0.908–1.373)^a^	0.297^a^
	Male	0.786 (0.588–1.049)^b^	0.102^b^	0.758 (0.545–1.053)^b^	0.098^b^
	Female	1.247 (0.998–1.558)^b^	0.052^b^	1.481 (1.127–1.948)^b^	0.005^b^

*SNP* single nuclear polymorphisms, *HR* hazard ratio, *CI* confidence interval, *P* probability

^a^Cox regression analysis adjusted for age, gender and BMI

^b^Cox regression analysis adjusted for age and BMI

### Quantitative trait analysis

Quantitative traits of FPG, insulin, HOMA indices and IRS1 were analyzed according to the genotype of MG53.

In Yixing population, a linear increase in insulin was observed with the variants of rs12929077 (P = 0.013) in untreated T2D cases, and the insulin concentration in the subjects with AA, AG and GG genotypes was 8.70 ± 8.05, 10.71 ± 11.16 and 13.41 ± 14.26 mU/L, respectively. Both HOMA-IR and HOMA-IS according to the rs12929077 genotype were significantly different, with P values of 0.02 and 0.023, respectively (Table 6). In the IFG group, AA genotype carriers of rs12929077 presented a relatively lower level of insulin and HOMA-IR and a higher HOMA-IS than those of the AG and GG genotypes.

**Table 6:** Comparison of FINS, FPG and HOMA indices according to the genotypes of *MG53* in Yixing population

Group	Variable	rs7186832			rs12929077		
		TT	TC	CC	AA	AG	GG
NGT	n	1836	910	99	1681	1019	127
	FINS (mU/L)	5.76 ± 4.38	5.75 ± 4.47	5.21 ± 3.90	5.77 ± 4.42	5.72 ± 4.42	5.29 ± 3.60
	FPG (mmol/L)	4.89 ± 0.59	4.90 ± 0.58	4.80 ± 0.61	4.90 ± 0.59	4.89 ± 0.58	4.87 ± 0.51
	HOMA-β	80.86 ± 309.33	85.29 ± 133.71	65.35 ± 110.37	80.18 ± 322.51	84.92 ± 128.53	71.46 ± 99.74
	HOMA-IR	1.26 ± 0.97	1.27 ± 1.03	1.13 ± 0.90	1.27 ± 0.98	1.26 ± 1.01	1.15 ± 0.80
	HOMA-IS	0.066 ± 0.099	0.066 ± 0.921	0.662 ± 0.096	0.06 ± 0.10	0.65 ± 0.86	0.65 ± 0.64
IFG	n	569	281	25	520	313	36
	FINS (mU/L)	7.12 ± 5.43	7.89 ± 7.30	6.80 ± 3.89	6.89 ± 4.86	8.22 ± 7.76*	6.33 ± 3.28
	FPG (mmol/L)	6.02 ± 0.34	6.07 ± 0.38	5.96 ± 0.34	6.02 ± 0.34	6.07 ± 0.37	5.95 ± 0.34
	HOMA-β	57.07 ± 43.80	61.48 ± 53.29	55.58 ± 33.25	55.07 ± 38.38	64.64 ± 59.11	51.18 ± 23.93
	HOMA-IR	1.91 ± 1.47	2.14 ± 2.07	1.81 ± 1.06	1.85 ± 1.33	2.22 ± 2.16*	1.69 ± 0.95
	HOMA-IS	0.038 ± 0.041	0.037 ± 0.047	0.034 ± 0.016	0.039 ± 0.042	0.036 ± 0.045	0.033 ± 0.017
T2D (untreated)	n	230	96	15	197	119	21
	FINS (mU/L)	8.99 ± 7.88	11.72 ± 13.88	13.75 ± 16.51	8.81 ± 8.05	10.66 ± 11.59*	14.29 ± 14.70
	FPG (mmol/L)	9.74 ± 3.14	9.73 ± 3.36	8.67 ± 1.86	9.72 ± 3.16	9.88 ± 3.39	8.88 ± 1.67
	HOMA-β	35.11 ± 39.20	48.84 ± 72.56	67.50 ± 94.10	35.31 ± 41.75	40.83 ± 51.27*	63.03 ± 78.67
	HOMA-IR	4.06 ± 4.05	5.09 ± 5.90	4.79 ± 5.24	3.93 ± 4.03	4.86 ± 5.44*	5.35 ± 5.18
	HOMA-IS	0.024 ± 0.028	0.020 ± 0.020	0.026 ± 0.037	0.026 ± 0.032	0.018 ± 0.017*	0.021 ± 0.021
T2D (treated)	n	104	44	8	95	53	8
	FINS (mU/L)	8.21 ± 6.01	10.23 ± 10.94	12.09 ± 14.30	8.47 ± 6.45	11.03 ± 13.38	8.88 ± 4.42
	FPG (mmol/L)	9.27 ± 3.56	8.80 ± 3.08	9.95 ± 2.93	9.03 ± 3.27	9.25 ± 3.69	10.01 ± 2.95
	HOMA-β	41.6847.68	52.81 ± 72.15	41.99 ± 53.95	45.12 ± 50.91	54.97 ± 88.74	30.30 ± 13.73
	HOMA-IR	3.52 ± 3.83	3.88 ± 4.11	5.25 ± 5.64	3.40 ± 3.31	4.50 ± 5.49	4.01 ± 4.15
	HOMA-IS	0.025 ± 0.029	0.026 ± 0.026	0.021 ± 0.021	0.25 ± 0.029	0.027 ± 0.026	0.016 ± 0.012

Comparison of FINS, FPG, HOMA-β, HOMA-IR, HOMA-IS between genotypes rs7186832 and rs12929077 were adjusted for age, gender and BMI by general linear regression; Significant P value (P < 0.05) was observed and marker by “*” for comparing AG to AA of rs12929077, a linear increase in insulin was observed with the variants of rs12929077 (P = 0.013) in untreated T2D subjects. FINS, HOMA-β, HOMA-IR, HOMA-IS are log-transformed for comparison

*NGT* normal glucose tolerance, *IFG* impaired fasting glucose, *T2D* type 2 diabetes, *n* number of subjects, *BMI* body mass index, *FPG* fasting plasma glucose, *FINS* fasting insulin, *HOMA* homeostasis model assessment, *IR* insulin resistance, *IS* insulin sensitivity

No statistically significant difference in IRS1 levels was observed among the different genotypes of rs7186832 and rs12929077 in each group or in the whole population (Additional file 1: Table S2). A correlation analysis showed that IRS1 was significantly correlated with FPG (r = 0.131, P = 0.03) in 275 randomly selected subjects; however, this correlation could not be replicated in the NGT or IFG subgroups or in the untreated T2D population (Additional file 1: Table S3). No statistical correlation was observed between IRS1 and FINS or HOMA-β, HOMA-IR and HOMA-IS (P > 0.05).

**Fig. 1:**
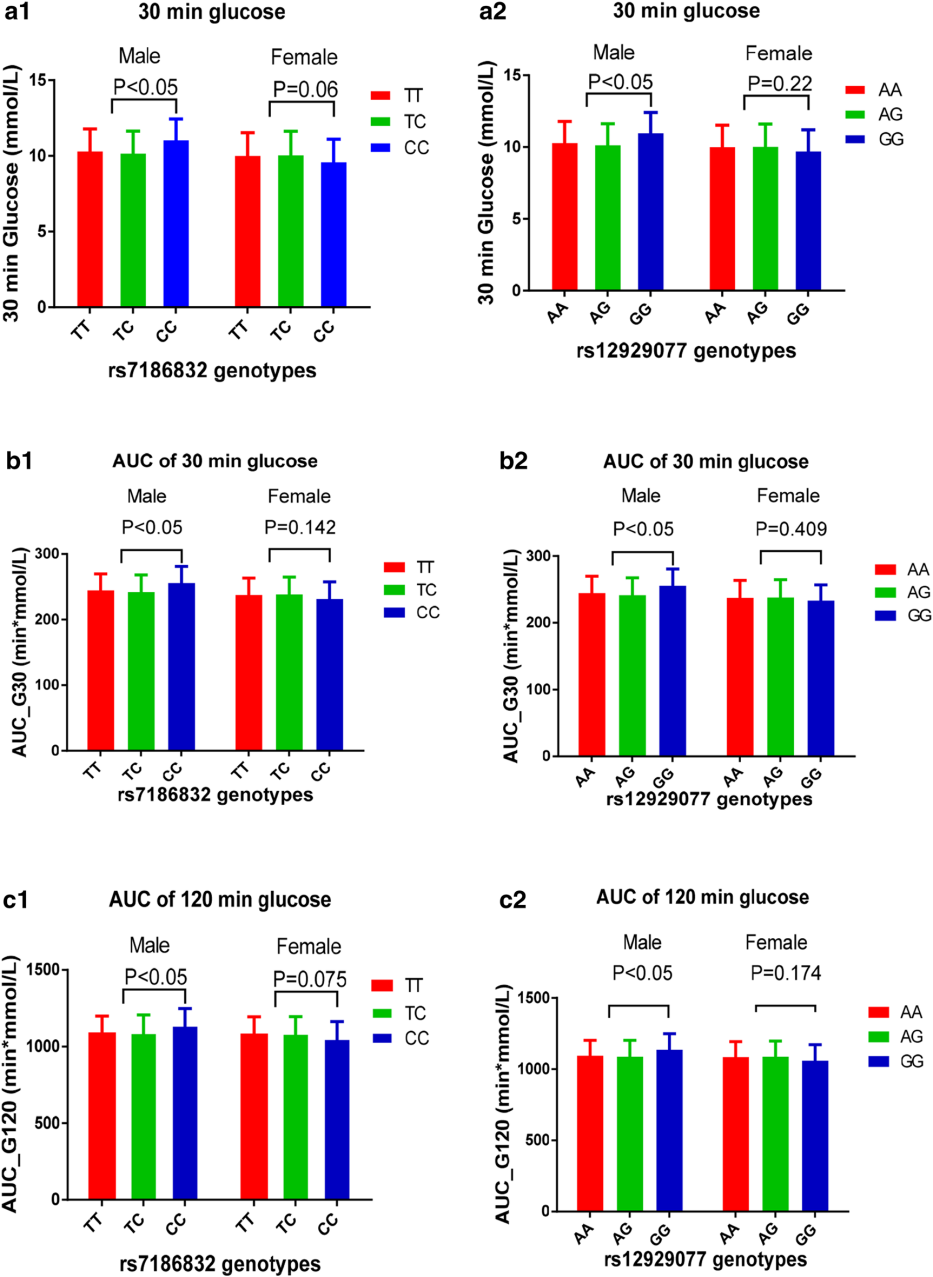
Stratification analysis showed that 30 min glucose (**a1**, **a2**), AUC of 30 min glucose (**b1**, **b2**) and AUC of 30 min glucose (**c1**, **c2**) after glucose load increase significantly in CC genotype (vs TT + TC) of rs7186832 carriers and GG genotype (vs AA + AG) of rs12929077 in males but not in females

In the Gulou population, no significant difference in glucose, insulin or the indices of OGTT and HOMA was detected among the genotypes of MG53 SNPs (Additional file 1: Table S4) after glucose loading. Further stratification analysis showed that the 30-min glucose, the area under the curve (AUC) of 30-min glucose and the AUC of 120-min glucose increased significantly with the CC genotype (vs TT + TC) of rs7186832 and the GG genotype (vs AA + AG) of rs12929077 in males but not in females (Fig. 1). The results of stratification analysis by gender are listed in Additional file 1: Table S5.

## Discussion

MG53, also known as tripartite motif 72 (TRIM72) in humans, is a newly identified member of the tripartite motif-containing (TRIM) family and is specifically expressed in the skeletal muscle and heart. The up-regulation of MG53 has been observed in high-fat diet (HFD)-induced obese mice, db/db diabetic mice, spontaneously hypertensive rats and non-human primate models of metabolic syndrome as compared to control animals. Ko et al. have confirmed that MG53 overexpression inhibits IRS1 phosphorylation and myogenesis in C2C12 myoblasts (Lee et al. 2010), and the insulin receptor and IRS1 levels notably changed when the insulin signal pathway is blocked. In this study, we observed positive association of *MG53* polymorphisms and IFG and T2D in females in Nantong rural population but not in Wuxi urban populations. In Yixing rural population of cohort study, the association of *MG53* polymorphisms and IFG was further replicated in females. These findings support that *MG53* variants might confer risk susceptibility to the development of T2D of females in rural population.

Furthermore, in Yixing rural population, FINS linearly increased with the variation of rs12929077 in the untreated T2D population, and differential HOMA-β, HOMA-IR and HOMA-IS were observed in both the IFG group and untreated T2D population. The above results verify the population-based evidence associating *MG53* with HOMA-IR, HOMA-IS, and T2D in the Han Chinese population. The findings from the present study thus confirm the role of MG53 in IR (Song et al. 2013). In addition, the genetic effects of *MG53* on islet beta cell secretion and regulating blood glucose function by OGTT were evaluated in Gulou urban population and the results indicated that the 30-min glucose, AUC of 30 min glucose and AUC of 120-min glucose increased with the variation of rs7186832 and rs12929077 in males but not in females. These findings provide further evidence strengthening the impact of *MG53* on the development of T2D.

We further evaluated a regional LD plot (http://www.broadinstitute.org/mpg/snap/ldplot.php) of the two positive SNPs in T2D (Additional file 1: Figure S1). The LD values (r^2^) were estimated for neighbouring loci and rs7186832 (r^2^ > 0.9) and rs12929077 (r^2^ > 0.8). We suggest that these closely linked loci need to be considered to further evaluate the genetic effect of *MG53* in T2D.

Although the SNP rs7186832 in exon 3 is a synonymous variant, an online bioinformatics prediction tool (http://snpinfo.niehs.nih.gov/cgi-bin/snpinfo/snpfunc.cgi) indicated that the rs7186832 C > T variant acts in splicing regulation (Exon Splicing Silencer, ESS) for MG53, and the rs12929077 G > A variant is associated with transcription factor binding sites (TFBS) to AP2α (core match score = 0.996) and BRCA (core match score = 0.994).

The AP2α transcription factor belongs to a family of three closely related nuclear proteins that regulate genes involved in development, apoptosis, and cell cycle control (Hilger-Eversheim et al. 2000). A previous study has reported that the AP2a site acts as a positive regulator on site 5 in SLC2A10, which encodes high-affinity glucose transporter 10 (GLUT10) (Segade et al. 2005). GLUT10 is widely expressed in adult tissues, including organs that play major roles in glucose homeostasis (Rothman et al. 1995), and the haplotype of SLC2A10 is modestly associated with T2D (Lin et al. 2006). BRCA (breast cancer, early onset) encodes a nuclear phosphoprotein that plays a role in maintaining genomic stability, and a previous study has reported that after a diagnosis of, women breast cancer with a BRCA1 or BRCA2 mutation face a twofold increase in the risk of diabetes (Bordeleau et al. 2011). These data provide considerable biologic plausibility for a role of MG53 in glucose homeostasis, insulin signal regulation and T2D.

This study did not identify the IRS1 level correlating with the variants of *MG53* or the HOMA index. Given the tissue specificity of MG53 and factors affecting the IRS1 level in plasma (Krutzfeldt et al. 2000; Chibalin et al. 2000), the plasma IRS1 level may only partly reflect MG53 expression. Meanwhile, this discrepancy may indicate that IRS1 might not directly interfere with the genetic effects of *MG53* on pancreatic β cell function, IR or T2D, and further research on IRS1 function is warranted.

Besides the potential bias in case–control studies, there are some limitations as follows. Owing to a lack of an appropriate ELISA kit and muscle tissues, plasma MG53 expression levels could not be detected; thus, correlations between *MG53* polymorphisms, MG53 expression and T2D risk could not be established in our study population. Regardless of the above limitation, this study provides updated evidence of *MG53* polymorphisms, HOMA indices and T2D. In case of potential type I error, further replication study in large sample size population would be warranted.

Conclusively, our study constitutes an initial examination to investigate whether *MG53* variants are associated with T2D, and the findings provide new insight into the molecular mechanism of MG53 involved in the pathogenesis of T2D through the effects on pancreatic β-cell function and IR.

## Additional file

**Additional file 1.** Supplementary material contains supplementary tables 1 to 5 and supplementary figure.
