# Supplementary material for: Evaluation of common variants in MG53 and the risk of type 2 diabetes and insulin resistance in Han Chinese
Source: Springerplus. 2016 May 12;5(1):612. doi: 10.1186/s40064-016-2218-1 (PMC5395513; doi:10.1186/s40064-016-2218-1)
Supplement: Supplementary file 1 — Additional file 1. Supplementary material contains supplementary tables 1 to 5 and supplementary figure. [file 40064_2016_2218_MOESM1_ESM.docx]

**Table S1 Association of MG53 genotypes with the risk of T2D from NGT and IFG in Yixing cohort population**

| SNPs | FPG levels | Gender | Additive model | |  | Dominant model | |
| --- | --- | --- | --- | --- | --- | --- | --- |
|  |  |  | HR(95%CI)^a^ | P^a^ |  | HR(95%CI)^a^ | P^a^ |
| rs7186832 | NGT+IFG | Male+Female | 1.178(0.888-1.564) | 0.256 |  | 1.167(0.836-1.628) | 0.364 |
|  |  | Male | 0.820(0.519-1.630) | 0.775 |  | 0.829(0.429-1.602) | 0.576 |
|  |  | Female | 1.286(0.927-1.75) | 0.132 |  | 1.322(0.896-1.952) | 0.16 |
|  | NGT | Male+Female | 1.015(0.608-1.693) | 0.956 |  | 0.898(0.491-1.643) | 0.728 |
|  |  | Male | 0.359(0.083-1.554) | 0.171 |  | 0.352(0.077-1.614) | 0.179 |
|  |  | Female | 1.276(0.731-2.228) | 0.391 |  | 1.140(0.580-2.242) | 0.704 |
|  | IFG | Male+Female | 1.254(0.889-1.769) | 0.197 |  | 1.292(0.863-1.933) | 0.213 |
|  |  | Male | 1.188(0.625-2.259) | 0.599 |  | 1.063(0.500-2.259) | 0.875 |
|  |  | Female | 1.291(0.858-1.945) | 0.221 |  | 1.406(0.871-2.270) | 0.164 |
| rs12929077 | NGT+IFG | Male+Female | 1.184(0.900-1.557) | 0.227 |  | 1.129(0.812-1.568) | 0.47 |
|  |  | Male | 0.972(0.569-1.663) | 0.918 |  | 0.841(0.445-1.590) | 0.595 |
|  |  | Female | 1.279(0.929-1.761) | 0.132 |  | 1.263(0.858-1.860) | 0.236 |
|  | NGT | Male+Female | 1.084(0.668-1.756) | 0.744 |  | 0.947(0.528-1.696) | 0.854 |
|  |  | Male | 0.481(0.141-1.645) | 0.244 |  | 0.487(0.132-1.802) | 0.281 |
|  |  | Female | 1.341(0.782-2.299) | 0.286 |  | 1.149(0.590-2.234) | 0.683 |
|  | IFG | Male+Female | 1.238(0.886-1.729) | 0.211 |  | 1.221(0.819-1.821) | 0.327 |
|  |  | Male | 1.173(0.634-2.171) | 0.61 |  | 0.964(0.459-2.025) | 0.964 |
|  |  | Female | 1.277(0.856-1.903) | 0.23 |  | 1.360(0.844-2.192) | 0.206 |
| ^a^ Cox regression analysis adjusted for age, gender and BMI. HR; Abbreviations: SNP, Single nuclear polymorphisms; FPG, Fasting plasma glucose; HR, Hazard ratio; CI, Confidence interval; NGT, Normal glucose tolerance; IFG, Impaired fasting glucose; P, Probability; T2D, Type 2 diabetes. | | | | | | | |

**Table S2 Comparison of IRS1 according to the genotypes of *MG53* in Yixing population**

| IRS1 | rs7186832 | | |  | rs12929077 | | |
| --- | --- | --- | --- | --- | --- | --- | --- |
|  | TT | TC | CC |  | AA | AG | GG |
| n | 158 | 84 | 33 |  | 122 | 99 | 50 |
| IRS1(pg/ml) | 1659±1498 | 1342±1146 | 1400±1086 |  | 1609±1485 | 1567±1314 | 1182±849 |
| Ln(IRS1) | 7.04±0.89 | 6.88±0.83 | 6.96±0.80 |  | 7.02±0.86 | 7.01±0.89 | 6.81±0.78 |

No statistical difference of Ln (IRS1) was detected according to the genotypes of rs7186832 and rs12929077 (P>0.05). Abbreviations: IRS1, Insulin receptor substrate 1; n, Number of subjects.

**Table S3 Correlation analysis of IRS1 and FPG, FINS, HOMA-β, HOMA-IR and HOMA-IS**

| Group | Pearson Correlation | FPG | FINS | HOMA-β | HOMA-IR | HOMA-IS |
| --- | --- | --- | --- | --- | --- | --- |
| NGT (n=65) | *r* | -0.197 | -0.01 | 0.04 | -0.01 | 0.15 |
|  | *P* | 0.116 | 0.97 | 0.77 | 0.94 | 0.22 |
| IFG (n=60) | *r* | -0.062 | 0.02 | 0.05 | 0.01 | -0.13 |
|  | *P* | 0.641 | 0.87 | 0.73 | 0.94 | 0.34 |
| T2D (untreated, n=150) | *r* | 0.012 | -0.13 | -0.12 | -0.09 | 0.08 |
|  | *P* | 0.885 | 0.13 | 0.14 | 0.28 | 0.32 |

No significant correlation between IRS1 and FPG, FINS, HOMA-Β, HOMA-IR, and HOMA-IS was observed in NGT, IFG and T2D subjects (P>0.05). Abbreviations: NGT, Normal glucose tolerance; IFG, Impaired fasting glucose; T2D, Type 2 diabetes; n, Number of subjects; IRS1, Insulin receptor substrate 1; FPG, Fasting plasma glucose; FINS, Fasting insulin; HOMA, Homeostasis model assessment; IR, Insulin resistance; IS, Insulin sensitivity.

| Variable | rs7186832 | | |  |  | rs12929077 | | | |
| --- | --- | --- | --- | --- | --- | --- | --- | --- | --- |
|  | TT | TC | CC | P value* |  | AA | AG | GG | P value* |
| n | 1233 | 607 | 90 |  |  | 1189 | 645 | 99 |  |
| I0(μU/ml) | 12.51±7.47 | 12.50±8.54 | 12.56±6.02 | 0.885 |  | 12.52±7.45 | 12.51±8.49 | 12.32±6.30 | 0.952 |
| I30(μU/ml) | 72.65±54.97 | 76.01±59.41 | 76.46±58.05 | 0.437 |  | 72.30±54.36 | 76.49±60.34 | 77.21±57.30 | 0.273 |
| I120(μU/ml) | 98.02±73.15 | 100.51±72.85 | 100.27±72.38 | 0.701 |  | 98.15±73.60 | 99.12±70.98 | 104.08±78.38 | 0.527 |
| G0(mmol/l) | 5.79±0.51 | 5.79±0.52 | 5.81±0.51 | 0.852 |  | 5.79±0.51 | 5.78±0.52 | 5.83±0.49 | 0.525 |
| G30(mmol/l) | 10.20±1.43 | 10.19±1.45 | 10.24±1.56 | 0.954 |  | 10.20±1.43 | 10.17±1.45 | 10.29±1.51 | 0.710 |
| G120(mmol/l) | 8.64±1.26 | 8.67±1.29 | 8.55±1.28 | 0.632 |  | 8.64±1.25 | 8.68±1.30 | 8.54±1.31 | 0.539 |
| AUC_I30(min*μU/ml) | 1278.11±875.25 | 1327.80±955.80 | 1335.34±919.52 | 0.481 |  | 1272.99±865.44 | 1334.08±969.84 | 1343.08±907.47 | 0.335 |
| AUC_I120(min*μU/ml) | 8962.05±5852.10 | 9265.42±6155.23 | 9288.06±6083.85 | 0.498 |  | 8946.87±5847.84 | 9231.80±6107.62 | 9501.81±6280.53 | 0.367 |
| AUC_G30(min*mmol/l) | 240.01±25.88 | 239.77±26.34 | 240.85±27.12 | 0.913 |  | 240.07±25.94 | 239.38±26.29 | 241.99±26.24 | 0.587 |
| AUC_G120(min*mmol/l) | 1088.25±107.81 | 1088.77±110.88 | 1086.84±121.33 | 0.976 |  | 1088.21±108.16 | 1087.84±110.69 | 1089.82±117.62 | 0.988 |
| IRI30 | 5.37±3.70 | 5.59±4.13 | 5.59±3.90 | 0.500 |  | 5.35±3.66 | 5.63±4.17 | 5.59±3.84 | 0.348 |
| IRI120 | 8.26±5.37 | 8.55±5.72 | 8.59±5.59 | 0.487 |  | 8.25±5.36 | 8.52±5.68 | 8.75±5.72 | 0.366 |
| IGI | 14.69±13.59 | 15.57±15.33 | 15.27±16.76 | 0.776 |  | 14.56±13.41 | 15.79±15.56 | 15.28±16.16 | 0.783 |
| HOMA-IR | 3.24±2.00 | 3.23±2.24 | 3.26±1.67 | 0.847 |  | 3.24±2.00 | 3.23±2.23 | 3.20±1.72 | 0.895 |
| HOMA-β | 112.84±69.78 | 114.63±89.18 | 112.78±54.61 | 0.921 |  | 112.82±69.48 | 115.05±88.46 | 109.57±57.54 | 0.882 |
| HOMA-IS | 0.019±0.018 | 0.019±0.013 | 0.018±0.015 | 0.794 |  | 0.018±0.017 | 0.019±0.012 | 0.019±0.016 | 0.746 |

**Table S4 Comparison of the indices of OGTT and HOMA according to the genotypes of MG53 in Gulou population**

*Comparing of the indices of OGTT and HOMA according to the genotypes of MG53 was adjusted for age, sex and BMI. I0, I30, I120, AUC_I30, AUC_I120, IRI30, IRI120, IGI, HOMA-β, HOMA-IR, HOMA-IS are log-transformed for comparison. Abbreviations: n, Number of subjects; BMI, Body mass index; I0, Fasting plasma insulin; I30, 30-min plasma insulin after glucose loading; I120, 120-min plasma insulin after glucose loading; G0, Fasting plasma glucose; G30, 30-min plasma glucose after glucose loading; G120, 120-min plasma glucose after glucose loading; AUC, Area under the curve; HOMA, Homeostasis model assessment; IR, Insulin resistance; IS, Insulin sensitivity.

**Table S5 Stratification analysis by gender for comparison of the indices of OGTT and HOMA according to the genotypes of *MG53* in Gulou population**

| Gender | Variable | rs7186832 | | |  |  | rs12929077 | | |  |
| --- | --- | --- | --- | --- | --- | --- | --- | --- | --- | --- |
|  |  | TT | TC | CC | P value* |  | AA | AG | GG | P value* |
| Male | n | 425 | 221 | 35 |  |  | 411 | 235 | 38 |  |
|  | Insulin0(μU/ml) | 12.50±8.25 | 12.52±10.08 | 13.73±7.03 | 0.657 |  | 12.54±8.31 | 12.50±9.91 | 13.30±6.92 | 0.842 |
|  | Insulin30(μU/ml) | 69.81±53.94 | 75.40±68.37 | 83.97±66.36 | 0.268 |  | 69.18±53.67 | 76.63±68.01 | 84.24±66.84 | 0.157 |
|  | Insulin120(μU/ml) | 89.06±66.23 | 94.73±73.49 | 105.13±78.14 | 0.268 |  | 88.83±66.21 | 93.21±72.78 | 108.63±77.88 | 0.193 |
|  | Glocose0(mmol/l) | 5.91±0.50 | 5.87±0.54 | 5.92±0.54 | 0.552 |  | 5.92±0.50 | 5.85±0.54 | 5.95±0.54 | 0.214 |
|  | Glocose30(mmol/l) | 10.39±1.39 | 10.25±1.40 | 11.13±1.32 | **0.002** |  | 10.40±1.4 | 10.24±1.39 | 11.08±1.34 | **0.002** |
|  | Glocose120(mmol/l) | 8.47±1.48 | 8.61±1.39 | 8.49±1.17 | 0.407 |  | 8.48±1.48 | 8.57±1.42 | 8.48±1.19 | 0.669 |
|  | AUCI30(min*μU/ml) | 1234.82±861.87 | 1318.83±1110.58 | 1465.65±1037.24 | 0.284 |  | 1225.98±858.75 | 1337.11±1103.02 | 1463.15±1039.43 | 0.196 |
|  | AUCI120(min*μU/ml) | 8384.47±5502.29 | 8947.97±6730.01 | 9989.23±6773.45 | 0.186 |  | 8337.20±5504.81 | 8980.41±6631.01 | 10142.48±6825.44 | 0.121 |
|  | AUCG30(min*mmol/l) | 244.67±25.03 | 242.01±26.14 | 255.89±25.21 | **0.009** |  | 244.89±25.06 | 241.51±26.02 | 255.61±24.94 | **0.004** |
|  | AUCG120(min*mmol/l) | 1093.74±105.96 | 1091.47±114.91 | 1138.89±109.29 | 0.053 |  | 1094.73±106.52 | 1088.59±113.46 | 1136.34±113.19 | **0.045** |
|  | IRI30 | 5.10±3.65 | 5.53±4.82 | 5.77±4.07 | 0.393 |  | 5.06±3.64 | 5.61±4.79 | 5.76±1.09 | 0.227 |
|  | IRI120 | 7.72±5.11 | 8.30±6.32 | 8.83±5.94 | 0.299 |  | 7.67±5.10 | 8.33±6.24 | 8.99±6.02 | 0.182 |
|  | IGR30 | 13.68±12.84 | 15.43±17.06 | 14.78±15.24 | 0.405 |  | 13.52±12.66 | 15.73±17.07 | 15.00±15.23 | 0.194 |
|  | HOMA-IR | 3.30±2.21 | 3.28±2.65 | 3.66±2.03 | 0.578 |  | 3.31±2.22 | 3.27±2.61 | 3.55±1.99 | 0.728 |
|  | HOMA-β | 107.47±77.63 | 113.00±112.30 | 115.227±54.83 | 0.776 |  | 107.48±78.24 | 113.69±110.01 | 110.78±54.87 | 0.780 |
|  | HOMA-IS | 0.019±0.023 | 0.019±0.013 | 0.019±0.022 | 0.936 |  | 0.019±0.023 | 0.019±0.013 | 0.019±0.021 | 0.892 |
| Female | n | 805 | 384 | 55 |  |  | 775 | 409 | 61 |  |
|  | Insulin0(μU/ml) | 12.51±7.04 | 12.50±7.54 | 11.81±5.21 | 0.910 |  | 12.50±6.97 | 12.52±7.59 | 11.71±5.86 | 0.845 |
|  | Insulin30(μU/ml) | 74.15±55.48 | 76.43±53.74 | 71.67±52.17 | 0.897 |  | 73.95±54.68 | 76.47±55.61 | 72.84±50.56 | 0.874 |
|  | Insulin120(μU/ml) | 102.84±76.47 | 104.06±72.47 | 96.97±68.99 | 0.939 |  | 103.18±76.91 | 102.72±69.89 | 101.25±79.19 | 0.955 |
|  | Glocose0(mmol/l) | 5.73±0.50 | 5.74±0.50 | 5.73±0.47 | 0.975 |  | 5.73±0.50 | 5.74±0.51 | 5.76±0.45 | 0.827 |
|  | Glocose30(mmol/l) | 10.10±1.43 | 10.15±1.48 | 9.68±1.43 | 0.060 |  | 10.10±1.43 | 10.13±1.48 | 9.80±1.41 | 0.220 |
|  | Glocose120(mmol/l) | 8.75±1.11 | 8.70±1.23 | 8.59±1.35 | 0.620 |  | 8.73±1.11 | 8.74±1.21 | 8.58±1.39 | 0.580 |
|  | AUC_I30(min*μU/ml) | 1300.09±882.12 | 1334.21±856.70 | 1252.42±835.53 | 0.911 |  | 1297.01±868.70 | 1334.97±886.73 | 1268.28±814.84 | 0.899 |
|  | AUC_I120(min*μU/ml) | 9265.21±6012.99 | 9443.36±5805.02 | 8841.85±5620.90 | 0.934 |  | 9268.33±6003.74 | 9386.21±5792.64 | 9102.71±5939.41 | 0.988 |
|  | AUC_G30(min*mmol/l) | 237.53±25.93 | 238.48±26.40 | 231.54±26.01 | 0.142 |  | 237.52±25.98 | 238.12±26.42 | 233.50±23.46 | 0.409 |
|  | AUC_G120(min*mmol/l) | 1085.84±108.09 | 1087.21±108.61 | 1053.71±108.81 | 0.075 |  | 1085.13±108.26 | 1087.65±109.22 | 1060.83±111.62 | 0.174 |
|  | IRI30 | 5.51±3.72 | 5.62±3.66 | 5.48±3.82 | 0.938 |  | 5.50±3.67 | 5.63±3.78 | 5.49±3.71 | 0.906 |
|  | IRI120 | 8.54±5.48 | 8.68±5.35 | 8.44±5.40 | 0.961 |  | 8.54±5.47 | 8.64±5.35 | 8.60±5.57 | 0.906 |
|  | IGR30 | 15.21±13.95 | 15.66±14.26 | 15.58±17.79 | 0.750 |  | 15.11±13.77 | 15.83±14.64 | 15.45±16.84 | 0.751 |
|  | HOMA-IR | 3.21±1.89 | 3.21±1.98 | 3.01±1.36 | 0.882 |  | 3.20±1.87 | 3.21±1.99 | 2.98±1.51 | 0.832 |
|  | HOMA-β | 115.67±65.26 | 115.71±72.97 | 111.21±54.91 | 0.967 |  | 115.66±64.30 | 115.96±73.72 | 108.81±59.57 | 0.854 |
|  | HOMA-IS | 0.018±0.014 | 0.018±0.012 | 0.018±0.008 | 0.782 |  | 0.018±0.014 | 0.019±0.012 | 0.018±0.009 | 0.815 |

*Comparing of the indices of OGTT and HOMA according to the genotypes of MG53 was adjusted for age and BMI. I0, I30, I120, AUC_I30, AUC_I120, IRI30, IRI120, IGI, HOMA-β, HOMA-IR, HOMA-IS are log-transformed for comparison. Abbreviations: n, Number of subjects; BMI, Body mass index; I0, Fasting plasma insulin; I30, 30-min plasma insulin after glucose loading; I120, 120-min plasma insulin after glucose loading; G0, Fasting plasma glucose; G30, 30-min plasma glucose after glucose loading; G120, 120-min plasma glucose after glucose loading; AUC, Area under the curve; HOMA, Homeostasis model assessment; IR, Insulin resistance; IS, Insulin sensitivity.

Supplementary figure

| 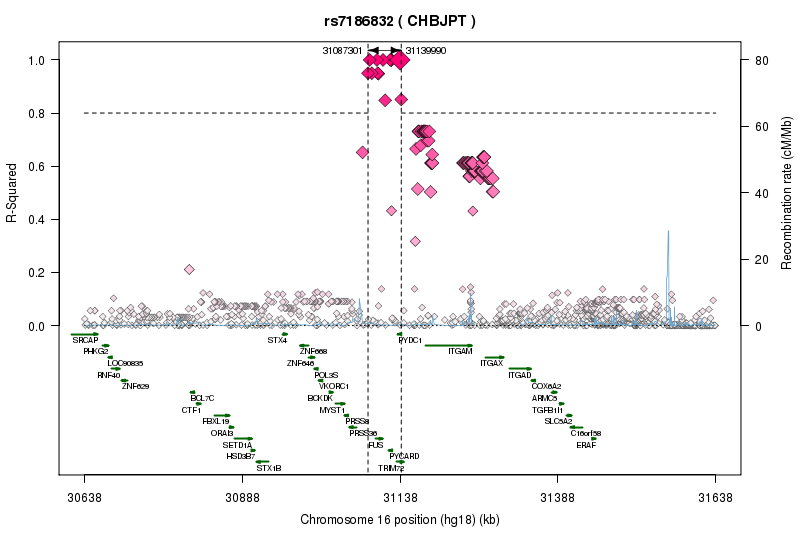 | 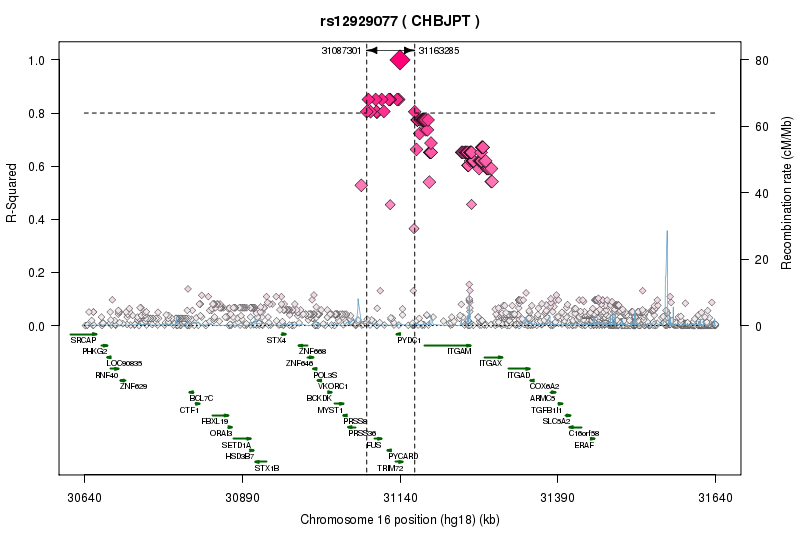 |
| --- | --- |
| **Supplementary Figure S1.** Regional LD Plots of rs7186832 and rs12929077 in *MG53.* | |
